# The Raine Syndrome Protein FAM20C Is a Golgi Kinase That Phosphorylates Bio-Mineralization Proteins

**DOI:** 10.1371/journal.pone.0042988

**Published:** 2012-08-10

**Authors:** Hiroyuki O. Ishikawa, Aiguo Xu, Eri Ogura, Gerard Manning, Kenneth D. Irvine

**Affiliations:** 1 Graduate School of Science, Chiba University, Chiba-shi, Chiba, Japan; 2 Howard Hughes Medical Institute, Waksman Institute and Department of Molecular Biology and Biochemistry, Rutgers, The State University of New Jersey, Piscataway, New Jersey, United States of America; 3 Razavi Newman Center for Bioinformatics, Salk Institute for Biological Studies, La Jolla, California, United States of America; National Institutes of Health (NIH), United States of America

## Abstract

Raine syndrome is caused by mutations in *FAM20C*, which had been reported to encode a secreted component of bone and teeth. We found that FAM20C encodes a Golgi-localized protein kinase, distantly related to the Golgi-localized kinase Four-jointed. *Drosophila* also encode a Golgi-localized protein kinase closely related to FAM20C. We show that FAM20C can phosphorylate secreted phosphoproteins, including both Casein and members of the SIBLING protein family, which modulate biomineralization, and we find that FAM20C phosphorylates a biologically active peptide at amino acids essential for inhibition of biomineralization. We also identify autophosphorylation of FAM20C, and characterize parameters of FAM20C’s kinase activity, including its Km, pH and cation dependence, and substrate specificity. The biochemical properties of FAM20C match those of an enzymatic activity known as Golgi casein kinase. Introduction of point mutations identified in Raine syndrome patients into recombinant FAM20C impairs its normal localization and kinase activity. Our results identify FAM20C as a kinase for secreted phosphoproteins and establish a biochemical basis for Raine syndrome.

## Introduction

The main structural component of bone is a composite of secreted extracellular proteins and the mineral hydroxyapatite. Insufficient bone density is a significant health concern for a majority of the human population as they age. Excess mineralization is also implicated in pathological conditions. Human genetic diseases can identify proteins that modulate biomineralization. Raine syndrome (lethal osteosclerotic bone dysplasia) is associated with increased ossification resulting in skeletal malformation [1], [2], [3]. Raine syndrome is caused by mutations in FAM20C [4], [5], [6], which has been reported to encode a secreted component of bone and teeth [7], [8].

Several phosphoproteins and phosphopeptides have been identified as having important roles in regulating biomineralization, including members of the Small Integrin-Binding Ligand N-linked Glycoproteins (SIBLING) protein family. These are a family of five secreted phosphoproteins, osteopontin (OPN), bone sialoprotein (BSP), dentin matrix protein 1 (DMP1), dentin sialophosphoprotein (DSPP) and matrix extracellular phosphoglycoprotein (MEPE), each of which contains an integrin binding motif [9], [10], [11], . They are highly expressed in bone and teeth, and have been implicated in modulating biomineralization through both genetic and biochemical studies. Moreover, their ability to modulate biomineralization can be affected by their phosphorylation status [9], [13], [14], [15], [16], [17].

The discovery of protein phosphorylation was first reported over a hundred years ago through characterization of the milk protein Casein [18]. The first description of an enzymatic activity that could phosphorylate proteins occurred over fifty years ago, using Casein as a substrate [19]. Two families of ubiquitously-expressed protein kinases have been termed Casein kinase 1 (CK1) and Casein Kinase 2 (CK2). However, they are unlikely to contribute to biological phosphorylation of Casein, as Casein is a secreted protein, whereas CK1 and CK2 are cytoplasmic and nuclear. A distinct enzymatic activity that could be responsible for endogenous Casein phosphorylation, termed Golgi casein kinase (G-CK) was first described in lactating mammary glands [20], but not molecularly identified. CK1, CK2, and G-CK all prefer acidic sequence motifs, but differ in their site preferences [21].

In earlier research, we identified *Drosophila* Four-jointed (Fj) as the first molecularly characterized Golgi-localized protein kinase [22]. Fj phosphorylates cadherin domains of the transmembrane receptor and ligand of the *Drosophila* Fat signaling pathway, Fat and Dachsous [22]. This phosphorylation of Fat and Dachsous modulates binding between [23], [24]. As Fj has little sequence similarity to known protein kinases, and was the first molecularly identified Golgi-localized protein kinase, it defined a new class of protein kinases.

Here we describe our identification of FAM20C as a Golgi-localized protein kinase related to Fj. FAM20C can phosphorylate secreted phosphoproteins, and characterization of its activity identifies FAM20C as a Golgi casein kinase. FAM20C substrates include phosphoproteins and peptides with known roles in regulating biomineralization, including Osteopontin and other members of the SIBLING protein family. Introduction of point mutations identified in human patients into recombinant FAM20C impairs its normal localization and kinase activity. Our results describe a novel kinase activity, and provide in vivo, genetic confirmation of the importance of secreted protein phosphorylation to the regulation of biomineralization.

## Results and Discussion

### Identification of Candidate Secretory Pathway Kinases

**Figure 1:**
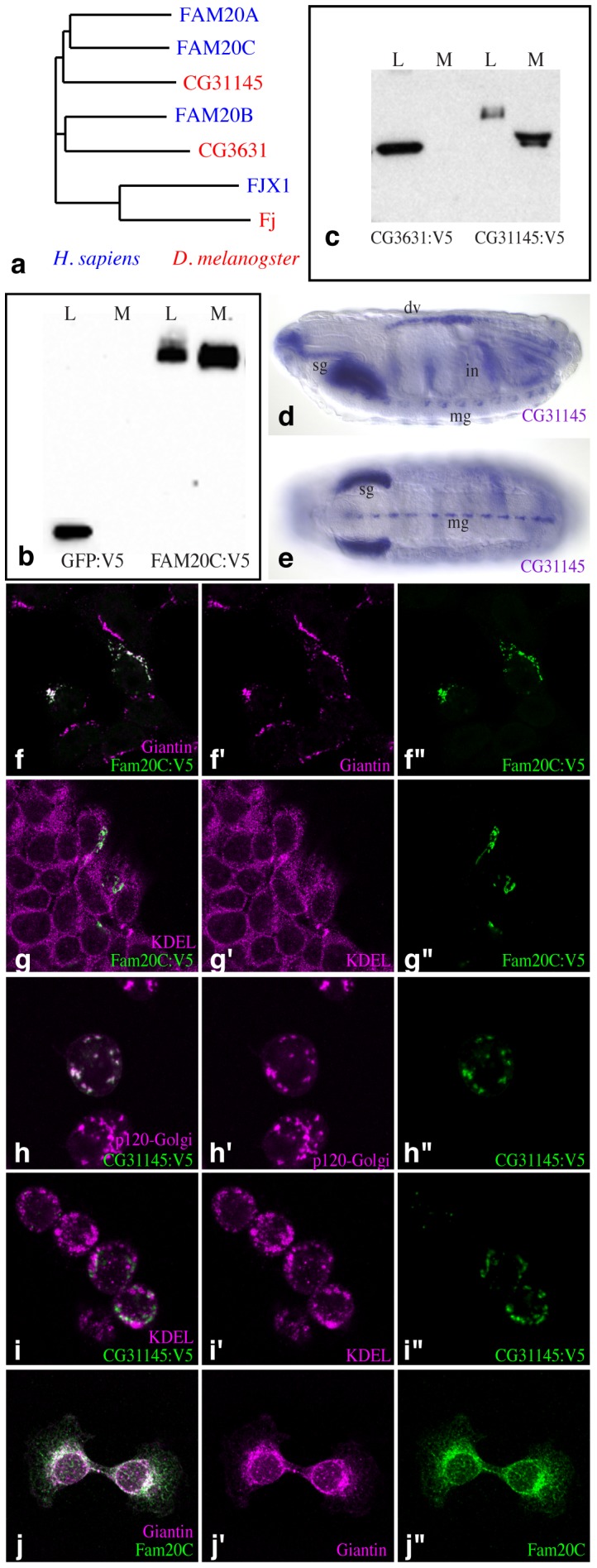
Localization of FAM20C proteins. a) Relative amino acid sequence similarity amongst Golgi kinases in flies (red) and humans (blue). Sequences compared were: *Drosophila* CG3631 (UniProt Q95T10), CG31145 (Q9VCK5), Four-jointed (Fj, P54360), human FAM20A (Q96MK3), FAM20B (O75063), FAM20C (Q8IXL6), and Four-jointed box protein 1 (Fjx1, Q86VR8). b) Western blot showing that FAM20C:V5 expressed in cultured cells is detected in both lysate (L) and medium (M), GFP:V5 serves as a non-secreted control. c) CG31145:V5 expressed in S2 cells is detected in both lysate (L) and medium (M), CG3631:V5 is only detected in lysate. d,e) in situ hybridization showing d) saggital view and e) ventral view of expression of *CG31145* in stage 16 *Drosophila* embryo; expression is detected in salivary gland (sg), dorsal vessel (dv), intestine (in), and midline glia (mg). Reproduced with permission from http://insitu.fruitfly.org/ [33]. f) Localization of FAM20C:V5 (green) in HEK293T cells overlaps a Golgi marker (Giantin, magenta). g) FAM20C:V5 localization is distinct from an ER marker (anti-KDEL, magenta). h) Localization of CG31145:V5 (green) in S2 cells overlaps a Golgi marker (p120-Golgi, magenta). i) Localization of CG31145:V5 (green) in S2 cells is distinct from an ER marker (anti-KDEL, magenta). j) Localization of FAM20C (green) in MC3T3 cells overlaps a Golgi marker (Giantin, magenta). For f–j, panels marked by prime symbols show single channels of the merged images to the left.

To identify other potential kinases related to Fj, we searched for protein sequences related to Fj and its mammalian homologue, Fjx1. The closest homologues are encoded by Family with sequence similarity 20 (FAM20), which in humans comprises *FAM20A*, *FAM20B*, and *FAM20C* [25]. Two members of this protein family were also identified in *Drosophila*, encoded by *CG31145*, which is most closely related to FAM20C and FAM20A, and *CG3631*, which is most closely related to FAM20B (Figs. 1a, S1). Sequence comparisons amongst family members in several species identified highly conserved amino acids shared by this protein family (Fig. S1). Bioinformatic analysis also suggested the presence of sequence motifs that are typical of all kinases, including amino acids implicated in catalysis and in binding metal ion co-factors (Fig. S1). Sequence analysis identified signal peptides for entry into the secretory pathway; if uncleaved these could form N-terminal type II transmembrane domains, which is a feature of Golgi-resident proteins. Together, these observations suggest that FAM20 proteins could encode kinases of the secretory pathway.

### Golgi Localization of FAM20 Proteins

We constructed V5-epitope tagged versions of murine FAM20 genes and expressed them in cultured HEK293T cells. Earlier studies of FAM20C (also known as DMP4) identified it as a secreted protein [7], [25]. However, some Golgi-resident transmembrane proteins, including glycosyltransferases [26], and the kinase Fj [27], are also secreted from cells, either by cleavage of a proteolytically sensitive stem between the transmembrane domain and the catalytic domain, or signal peptide cleavage. Western blotting on cell lysates and conditioned media of transfected cells revealed that FAM20C was present both in the medium and cell lysate, whereas FAM20A and FAM20B were only detected in the cell lysate (Figs. 1b, S2h). Immunolocalization revealed that FAM20C and FAM20B exhibited extensive overlap with a Golgi marker (Giantin), identifying it as a Golgi-localized protein (Figs. 1f, S2a). FAM20A exhibited substantial overlap with Giantin, but also weaker overlap with an endoplasmic reticulum (ER) marker (KDEL), which suggests that it is predominantly Golgi-localized, but also partially ER localized (Fig. S2c, d). In contrast, the subcellular distributions of FAM20B and FAM20C were clearly distinct from ER (Figs. 1g, S2b).

In *Drosophila* cells, transfected CG31145 protein overlapped a *Drosophila* Golgi marker (p120-Golgi) and was also secreted from cells, similar to FAM20C, to which it is most closely related (Fig. 1c, h). CG31145 localization was clearly distinct from an ER marker (Fig. 1i). In contrast, CG3631 overlapped both ER and Golgi markers, and was not detectably secreted from S2 cells (Figs. 1c, S2e, f). The distinct localization profiles of these proteins suggest that they have distinct functions. For FAM20C, we also confirmed the Golgi localization of endogenous protein by immunostaining of an osteoblast cell line (Figs. 1j, S2g).

### Expression of FAM20C Proteins

Expression profiling [28] shows that FAM20C is broadly expressed, but upregulated in bone and teeth, lactating mammary gland, and several other secretory tissues [29], [30]. Intriguingly, given the linkage of FAM20C to Raine syndrome, expression of FAM20C orthologues correlates with biomineralization in some species, including chicken, where it is upregulated during egg formation [31], and sea urchin, where it is upregulated during tooth development [32]. However, in *Drosophila*, *CG31145* expression does not appear to be associated with biomineralization. In embryos, it is prominently expressed in midline glia, salivary gland, intestine, and dorsal vessel (heart) (Fig. 1d, e) [33], [34].

### FAM20C is a Protein Kinase

**Figure 2:**
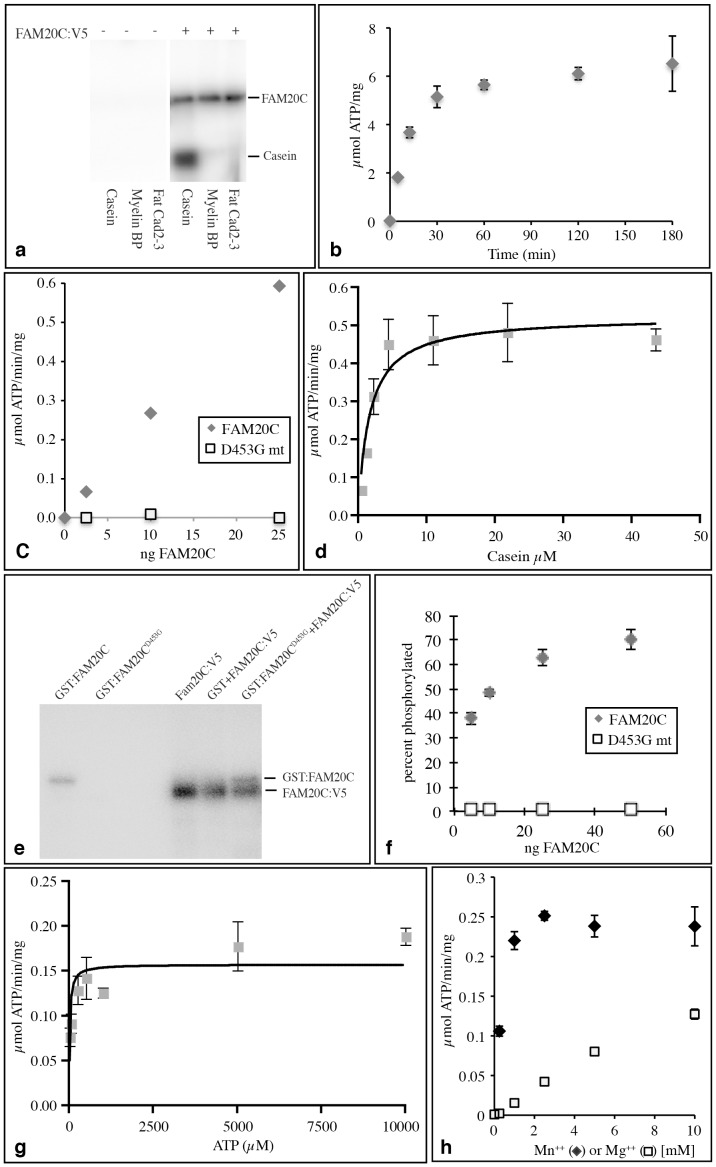
Kinase activity of FAM20C. a) Autoradiogram on protein gel showing the results of in vitro kinase assays in the presence (+) or absence (−) of FAM20C:V5 and the proteins indicated at bottom as substrates; the mobilities of FAM20C and Casein are indicated. Panels b−d,g,h show the results of kinase assays using dephosphorylated alpha casein as a substrate, and affinity purified FAM20C:V5 as the enzyme. b) Time course of FAM20C kinase reaction. c) Relationship between kinase activity and amount of wild-type or D453G mutant FAM20C:V5. d) Relationship between kinase activity and amount of Casein, curve fitting (using Graphpad Prism software) was used to determine Km. e) Autophosphorylation of FAM20C, as shown by autoradiogram on protein gel showing the results of in vitro kinase assays with the indicated proteins. A GST:FAM20C fusion protein expressed in bacteria is phosphorylated in a bacterial lysate, whereas an isoform with the D453G mutation is not. FAM20C:V5 can autophosphorylate, and FAM20C:V5 can phosphorylate GST:FAM20C with the D453G mutation. f) Autophosphorylation of FAM20C is concentration dependent, as the fraction of FAM20C phosphorylated increases at higher enzyme concentrations, consistent with a bimolecular reaction. Reactions were conducted in 10 µL volume, using 5, 10, 25, or 50 ng FAM20C. g) Relationship between kinase activity and amount of ATP, curve fitting (using Graphpad Prism software) was used to determine Km. h) Dependency of kinase activity on divalent cation concentration.

To examine FAM20C for kinase activity, we first assayed FAM20C:V5 secreted into the medium. Many protein kinases, including Fj, can undergo an autophosphorylation reaction. Thus, we incubated conditioned medium from FAM20C:V5-expressing HEK293T cells with radiolabeled ATP. This revealed that FAM20C:V5 was phosphorylated (not shown), suggesting that it encodes a kinase. To further characterize this apparent kinase activity, we took advantage of the V5-epitope tag to affinity-purify FAM20C:V5 from conditioned medium, using anti-V5 agarose beads, and then used this affinity purified protein as an enzyme source for in vitro kinase assays. As one candidate FAM20C substrate, we used a dephosphorylated form of alpha-Casein, as Casein is normally an abundant secreted phosphoprotein in milk. Dephosphorylated Casein was phosphorylated by affinity-purified FAM20C in vitro, as was FAM20C itself, establishing FAM20C as a Casein kinase (Fig. 2a). *Drosophila CG31145* also has Casein kinase activity (not shown). Conversely, when we examined Myelin basic protein, a common substrate for kinases that recognize basic sequence motifs, or cadherin domains of Fat that are substrates for Fj, phosphorylation by FAM20C was not detected (Fig. 2a). Thus, FAM20C is a Golgi-localized protein kinase with a substrate-specificity distinct from that of Fj.

### Characterization of FAM20C Kinase Activity

Using Casein as a model substrate, we characterized parameters of FAM20C kinase activity. Mutation of a conserved Asp near the catalytic center (D453G) abolished kinase activity, confirming that FAM20C, rather than any co-purifying contaminant, possesses kinase activity (Fig. 2c). This conclusion was further supported by detection of autophosphorylation of a GST:FAM20C fusion protein expressed in bacteria (Fig. 2e). By combining wild-type and D453G mutant forms of GST:FAM20C with FAM20C:V5, we were also able to determine that FAM20C autophosphorylation involves an intermolecular kinase reaction, because GST:FAM20C^D453G^ lacks autophosphorylation activity, but could be phosphorylated by FAM20C:V5 (Fig. 2e). Moreover, the fraction of FAM20C phosphorylated increased at higher concentrations of FAM20C, consistent with a bimolecular reaction (Fig. 2f). This contrasts with Fj, which undergoes a unimolecular autophosphorylation reaction [22].

Using Casein as a substrate, and under linear reaction conditions (5 minute assays, Fig. 2b), and averaging the results from several independent experiments, we obtained a Michaelis constant (*K*m) of FAM20C for Casein of 1.5 µM, a *K*m for ATP of 78 µM, a turnover number (kcat) of 52 per minute, and a V_max_ of 0.7 µM ATP/min/mg FAM20C (Fig. 2d, g and data not shown). These observations provide a biochemical description of FAM20C’s enzymatic activity, and confirm that it acts catalytically. Although most kinases have higher activity with Mg^2+^ as a cofactor, FAM20C exhibits higher activity with Mn^2+^ as a cofactor (Fig. 2h), similar to the Golgi-associated kinase Fj [22]. FAM20C is active over a wide pH range, but has slightly higher activity at or below pH 7.0 (Fig. S3a). FAM20C has been identified as a Ca^2+^-binding protein [7], but the presence or absence of Ca^2+^ did not significantly affect its kinase activity (Fig. S3b).

Golgi casein kinase (G-CK) was first described in lactating mammary glands [20], but not molecularly identified. Nonetheless, G-CK has been enzymatically characterized. Intriguingly, the *K*m for ATP of FAM20C (78 µM) is similar to that originally measured for G-CK (80 µM) [20]. The Km for Casein is higher (12 µM for G-CK) [20], but this could reflect differences in assay conditions, or in the quality or purity of this substrate. G-CK also exhibits a preference for Mn^++^ over Mg^++^ as a cofactor [35]. Thus, beyond their shared Golgi localization and ability to phosphorylate Casein, FAM20C and G-CK appear biochemically similar.

**Figure 3:**
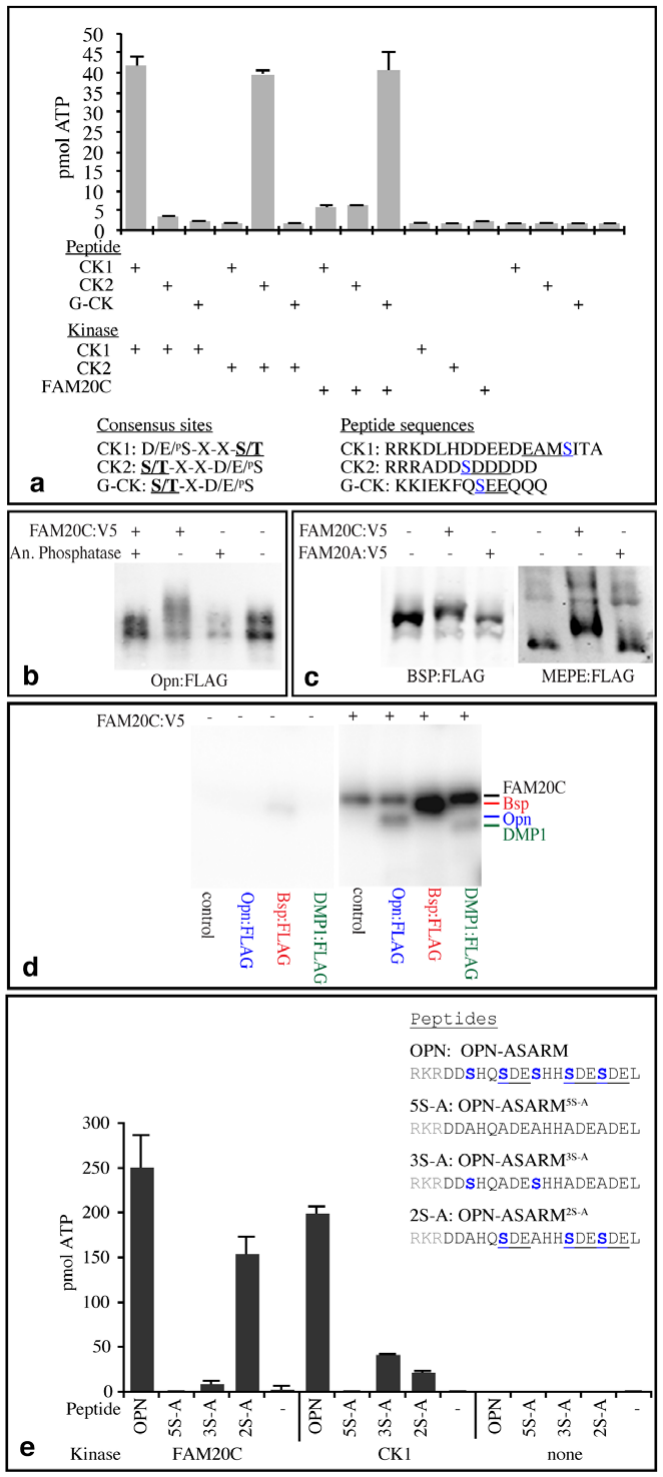
FAM20C phosphorylates SIBLING proteins. a) Activity of FAM20C on peptides reported to be uniquely recognized by G-CK, CK1, or CK2 (23). Phosphocellulose assays were performed using 100 µm of the indicated peptides, and 5 ng FAM20C, 5 units CK1, or 1 unit CK2, as indicated. Phosphorylation sites are underlined, with the Ser in blue. b) Western blot (anti-FLAG) showing mobility shift of Opn:FLAG induced by co-transfection of FAM20C:V5 (as indicated by +) in HEK293T cells, and its reversal by Antarctic phosphatase (An. phosphatase). c) Western blots (anti-FLAG) showing mobility shifts of Bsp:FLAG and MEPE:FLAG induced by co-transfection of FAM20C:V5, but not FAM20A:V5 (as indicated by +), in HEK293T cells. d) Autoradiogram on protein gel showing the results of in vitro kinase assays in the presence (+) or absence (−) of FAM20C:V5 and the proteins indicated at bottom as substrates; the mobility of FAM20Cis indicated. The substrate proteins were affinity purified on FLAG beads, mock purification of conditioned medium from untransfected cells was used as a negative control. The mobilities of labeled protein bands are indicated at right. e) Results of kinase assays using the indicated OPN-ASARM peptides (− indicates no peptide), and using the indicated kinases. The names and sequence of peptides used are indicated at top right. First three amino acids (gray) were added to enable assays using phosphocellulose paper. Ser residues are highlighted in blue and G-CK consensus sites are underlined.

To further explore the biochemical similarity of FAM20C to G-CK, we investigated their activity on model peptide substrates. CK1, CK2, and G-CK all prefer acidic sequence motifs, but differ in their site preferences [21]. Three peptides have been reported that were uniquely phosphorylated either by G-CK, CK1, or CK2 [21]. When we compared the relative activity of FAM20C, CK1, and CK2 on these peptides, we found that FAM20C exhibited a strong activity on the G-CK peptide, and only very weak activity on the other peptides, further supporting its identification as a protein responsible for G-CK activity (Fig. 3a).

Together with its normal Golgi localization, and other enzymatic properties, these observations identify FAM20C as a G-CK. Although G-CK was first described as an enzymatic activity almost 40 years ago [20], its molecular identity had remained elusive, and FAM20C is the first G-CK to be molecularly identified. As we were unable to detect G-CK activity in association with either FAM20A or FAM20B (not shown), it’s possible that FAM20C is the sole mammalian G-CK.

### SIBLING Proteins are FAM20C Substrates

The osteosclerotic bone dysplasia in Raine syndrome patients identifies a role for FAM20C in regulating bone formation. Given the established roles of SIBLING proteins in regulating biomineralization, and their identity as secreted phosphoproteins, they were obvious candidate substrates of FAM20C, especially as their ability to modulate biomineralization can be affected by phosphorylation [9], [13], [14], [15], [16], [17]. Moreover, the majority of phosphorylation sites identified in OPN conform to a consensus sequence for G-CK (S/T-X-D/E/^P^S) [36], and G-CK activity can phosphorylate OPN [37].

To investigate whether SIBLING proteins are substrates of FAM20C kinase activity, we co-expressed FLAG epitope-tagged OPN, BSP or MEPE together with FAM20C:V5 in cultured HEK293T cells. Co-expression of FAM20C, but not FAM20A, decreased their mobility on SDS-PAGE gels (Fig. 3b, c), consistent with an increase in their phosphorylation. Incubation of FAM20C-modified OPN:FLAG with phosphatase reversed this mobility shift (Fig. 3b). Thus, phosphorylation of SIBLING proteins can be promoted by FAM20C *in vivo*. To confirm that this involves a direct phosphorylation of SIBLING proteins by FAM20C, we performed in vitro kinase assays on three different affinity purified SIBLING proteins. Transfer of ^32^P onto OPN, BSP, and DMP1 was observed in the presence of FAM20C:V5, indicating that FAM20C can directly phosphorylate SIBLING proteins (Fig. 3d).

To further investigate the activity of FAM20C on a biologically relevant substrate, we assayed phosphorylation of a peptide including amino acids 115–132 of human OPN (OPN-ASARM, for acidic, serine- and aspartate-rich motif) [13]. ASARM peptides are found in several SIBLING proteins, and play key roles in regulating biomineralization. In its phosphorylated form, the OPN-ASARM peptide functions as an inhibitor of mineralization through binding to hydroxyapatite [13]. It contains 5 serine residues, three of which, when phosphorylated, are essential for inhibition of biomineralization [13]. Intriguingly, these three Ser residues are found within an SDE sequence motif, which conforms to the G-CK consensus (Fig. 3a, e). *In* *vitro* kinase reactions confirmed that OPN-ASARM is a FAM20C substrate, whereas a derivative with all five Ser residues replaced by Ala (OPN-ASARM^5S-A^) was not phosphorylated (Fig. 3e). A peptide in which only the three G-CK consensus sites were changed to Ala (OPN-ASARM^3S-A^) was phosphorylated much less effectively, whereas a derivative in which the other two Ser residues were changed to Ala (OPN-ASARM^2S-A^) was still efficiently phosphorylated by FAM20C (Fig. 3e). CK1 also phosphorylated OPN-ASARM, but by contrast to FAM20C, had similarly reduced levels of activity on both OPN-ASARM^3S-A^ and OPN-ASARM^2S-A^. Together, these observations indicate that FAM20C phosphorylates a biologically active peptide at specific amino acids that conform to G-CK consensus sites, and which are known from mutagenesis studies to be essential for the ability of this peptide to inhibit biomineralization [13]. This discovery also suggests a molecular explanation for Raine syndrome, as the increased bone mass associated with this disease could be explained by decreased phosphorylation of proteins and peptides that inhibit biomineralization, like OPN-ASARM.

### Raine Syndrome Mutations Impair FAM20C Kinase Activity

**Figure 4:**
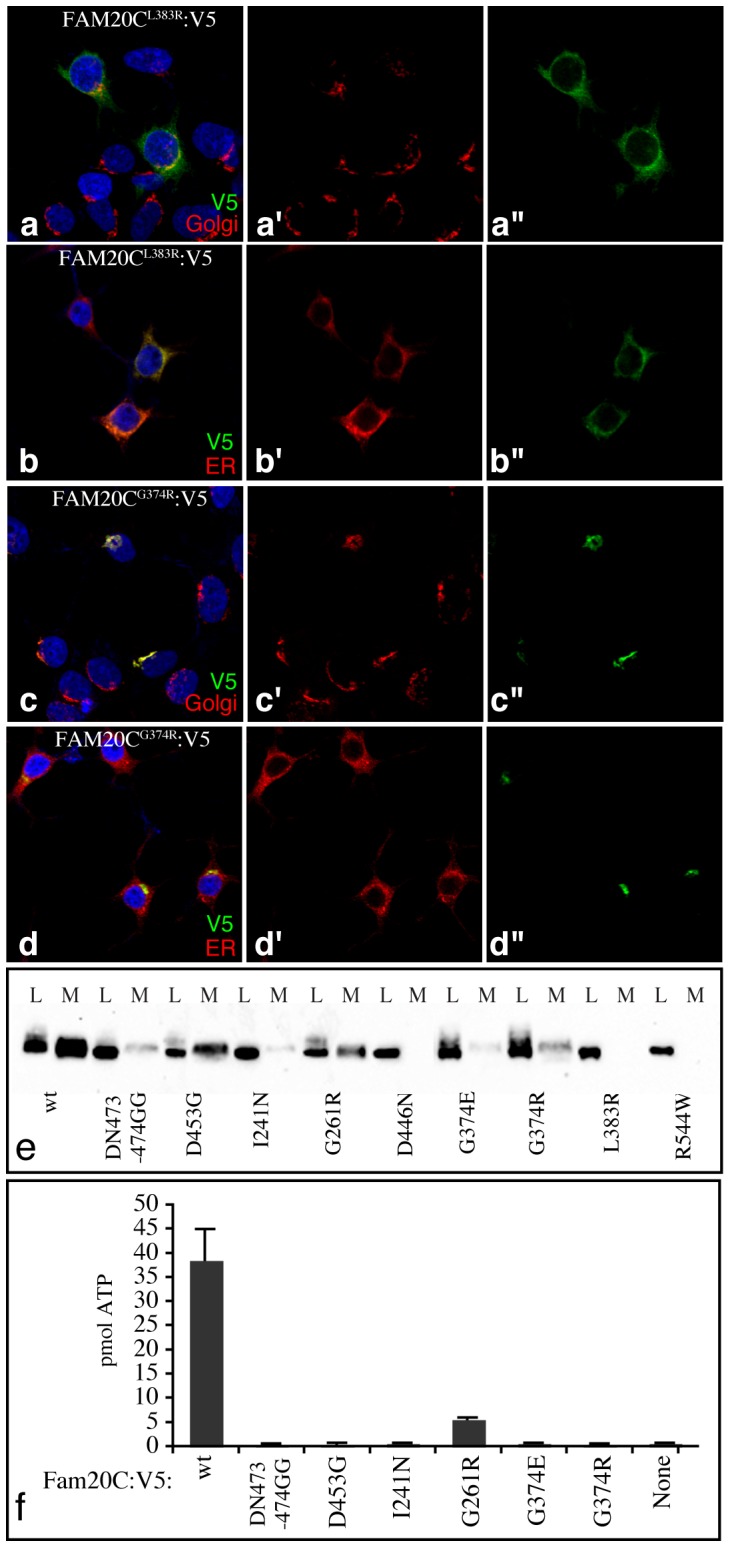
Localization and activity of FAM20C mutant isoforms. A–d Examples of localization of the indicated V5-tagged FAM20C mutant proteins (green) transfected into 293 cells, as compared to a Golgi markers (Giantin, a,c, red) or an ER marker (anti-KDEL, b,d red). Nuceli are labeled by DAPI (blue). Panels marked by prime symbols show single channels of the merged image to the left. e) Western blot showing localization of FAM20C mutant proteins in the lysate (L) and/or medium (M) of 293 cells. f) Results of kinase assays using the indicated FAM20C mutant proteins and the OPN-ASARM peptide as a substrate.

The inference that FAM20C modulates bone formation by acting as a protein kinase implies that mutations identified in human Raine syndrome patients should impair FAM20C kinase activity. To examine this, we engineered such mutations (corresponding to mouse FAM20C G374R, G374E, L383R, R544W, I241N, G261R, D446N, Fig. S4) into recombinant mouse FAM20C:V5 expressed in cultured cells. For comparison we also examined FAM20C with a mutation in the conserved Asp residue described above (D453G), and FAM20C with a mutation in the highly conserved “DFG” motif of protein kinases (Fig. S1) required for binding metal ion cofactors (DN473–474GG) (Fig. S4). These mutations in conserved motifs abolished catalytic activity without disrupting FAM20C Golgi localization, although they did reduce its secretion (Figs. 4, S5). Three of the mutations identified in patients (L383R, R544W, and D446N) resulted in mislocalization of protein from the Golgi to the ER, and an absence of detectable protein secreted into the media (Figs. 4, S5). Since misfolded secretory pathway proteins are typically retained in the ER, these observations suggest that these mutations result in misfolded protein, and we did not attempt to purify them. The remaining four mutant isoforms from patients were Golgi localized and were secreted into the medium, though less efficiently than wild-type FAM20C (Figs. 4, S5). Three of these (G374E, G374R, and I241N) had no detectable kinase activity, whilst the fourth (G261R) had diminished kinase activity (Fig. 4f). Thus, all four point mutations that did not appear to be associated with gross misfolding of FAM20C protein impaired its kinase activity, supporting the importance of kinase activity to FAM20C function in vivo. The G261R mutation in humans is associated with a non-lethal form of Raine syndrome [6], whereas homozygosity for G374 mutations was found in a lethal form of this disease [5]. Together, these observations suggest that the extent of impairment of kinase activity correlates with disease severity.

### Conclusions

Although the existence of G-CK was implied by the initial discovery of Casein phosphorylation over a hundred years ago on [18], and the discovery of an enzymatic activity that phosphorylates casein almost 60 years ago [19], the molecular identify of G-CK had proved elusive. Our observations, together with the recent report of Tagliabracci et al. [38], support the identification of FAM20C as G-CK. Our combined results also indicate that FAM20C kinase activity is essential for limiting bone formation in humans, and identify SIBLING proteins as an important class of FAM20C substrates. Since SIBLING proteins can act as nucleators or inhibitors of biomineralization depending upon their phosphorylation state [9], [12], [13], [14], [15], [16], [17], the increased bone density of Raine syndrome patients could be accounted for by decreased phosphorylation of SIBLING proteins, and their ASARM peptide products, which are known to inhibit mineralization when phosphorylated [13].

A recent study reported a gene-targeted mutation in murine FAM20C [39]. Rather than exhibiting the osteosclerosis characteristic of Raine syndrome, these mice exhibit hypophosphatemic Rickets, which includes decreased bone mass. The authors attribute this to affects on differentiation of osteogenic cells. However, SIBLING proteins have complex effects on bone formation [9], [13], [14], [15], [16]. OPN is generally thought of as an inhibitor of biomineralization [13], [40], but mutation of murine DMP1 causes hypophosphatemic Rickets, implicating it as a promoter of biomineralization [41], [42]. In vitro, phosphorylation of DMP1 affected crystal morphology rather than simply promoting or inhibiting mineralization [17]. Considering that DMP1 phosphorylation is promoted by FAM20C, lack of SIBLING protein phosphorylation might also contribute to the murine *FAM20C* phenotype. The distinct phenotypes of *FAM20C* mutations in humans and mice might thus reflect differences in how bone promoting and bone inhibiting activities of secreted phosphoproteins are balanced between these species.

Another important question for future studies is whether there exist additional protein kinases for secreted phosphoproteins, and if so whether any of them can provide G-CK activity. An analysis of the secreted phosphoproteome shows that most phosphorylated peptides include G-CK sites [43], which implicates G-CK as the main kinase for secreted proteins. Since we found that FAM20C has weak activity on peptides whose only Ser residue matches classic CK1 and CK2 sites, rather than the G-CK site, it is possible that phosphorylation in vivo of sites that don’t match the G-CK consensus is also carried out by FAM20C. Fj clearly has a distinct kinase specificity, which is limited to cadherin domains. FAM20B has been identified as a kinase that phosphorylates xylose within the glycosaminoglycan core linker [44]. This supports the general concept that FAM20 proteins are Golgi-localized kinases, but also reveals that they can have quite distinct substrate specificities. Thus, it remains an open question whether FAM20A, or any other proteins besides FAM20C, possess G-CK activity.

## Methods

### Cell Culture and Biochemistry

Human embryonic kidney (HEK293T) cells were grown in DMEM/F12 medium (Invitrogen) containing 10% fetal bovine serum. Lipofectamine (Invitrogen) was used for transfection of 1 µg of DNA into 5 mL culture. After 2 to 4 days induction, conditioned media (5 mL) was collected and centrifuged (3000 rpm, 15 min).

For secretion assays, conditioned media and cell lysates were subjected to Western blotting to detect FAM20C:V5, FAM20A:V5, and FAM20B:V5. GFP:V5 was used as a non-secreted control protein.

For mobility shift assays on OPN:FLAG, BSP:FLAG, DMP1:FLAG, DSPP:FLAG and MEPE:FLAG, conditioned media were subjected SDS-PAGE, and OPN:FLAG, BSP:FLAG, DMP1:FLAG, DSPP:FLAG and MEPE:FLAG were detected by Western blotting. Phosphatase treatments to reverse mobility shifts were performed using Antarctic phosphatase (New England Biolabs).

For FAM20C:V5 purification, pcDNA-FAM20C:V5 was transfected to HEK293T cells, and FAM20C:V5 was purified from conditioned media using anti-V5 agarose beads (Sigma). Conditioned medium was incubated with anti-V5 affinity gel (Sigma) overnight at 4°C. Agarose beads were collected by centrifugation, and washed 5 times with TBS. Proteins were eluted using V5 peptides in TBS (Sigma, final concentration: 100 µg/ml). For purification of OPN:FLAG, BSP:FLAG, DMP1:FLAG, DSPP:FLAG and MEPE:FLAG, conditioned media were incubated with anti-FLAG M2 affinity gel (Sigma) for over night at 4°C. Agarose beads were collected by centrifugation, and washed 5 times with TBS. Quantification was performed on gels stained by SYPRO Ruby Protein Gel Stain (Invitrogen), using BSA as a standard.

S2 cells (*Drosophila* Genomics Resource Center) were grown in Schneider’s Drosophila medium (Invitrogen) containing 10% fetal bovine serum. Cellufectin (Invitrogen) was used for transfection. 1 µg of UASattB-CG31145:V5 or UASattB-CG3631:V5 and 0.5 µg of pWAGal4 (a kind gift from Y. Hiromi) were co-transfected into 5 mL cell culture. After 2 days induction, conditioned media (5 mL) and cell lysate were collected.

### Western Blotting

HRP-conjugated mouse anti-V5 (1∶10,000, Invitrogen), and HRP-conjugated mouse anti-FLAG M2 (1∶10,000, Sigma) were used for Western blotting. Proteins were transferred to nitrocellulose membranes (Bio-Rad Laboratories) and detected using Super Signal West Dura (Pierce).

### Kinase Assays

FAM20C kinase assays were performed in 10 µl reactions, with 500 µM ATP, including 3 µCi (0.05 µM) of [gamma-^32^P]ATP (6000 Ci/mmol, PerkinElmer), 50 mM Tris (pH 7.0), 10 mM MnCl2, 0.1% BSA, purified FAM20C:V5 (5 ng, 0.066 pmol), and dephosphorylated alpha-Casein (0.1 µg, Sigma). The kinase reaction was linear at 5 minutes, and the reaction rate was dependent on enzyme and substrate concentrations. Reactions were stopped by adding SDS sample buffer and boiling, and one half of the reaction volume was subjected to SDS-PAGE. Radioactivity within the gels was detected using a Molecular Dynamics Phosphor Imager. For quantitation of gamma-^32^P incorporation, bands were cut out of the gels using the Phosphor Imager pictures as a reference, and then counted by liquid scintillation using a Beckman Coulter LC6500. Myelin Basic Protein (0.5 µg, New England Biolabs) and affinity-purified Fat2–3:FLAG (0.1 µg) [22] were used as control substrates. To determine pH-dependency, the pH in the reaction buffer was varied using Tris-HCl buffers. Synthesized peptides were designed from the sequence of human Osteopontin 115-Asp to 132-Leu, adding three basic amino acids (Arg-Lys-Arg) to its N-terminus to enable peptide binding to phosphocellulose paper (P81, Upstate). Peptides were purchased from Peptide2.0. Peptide1, OPN-ASARM (RKRDDSHQSDESHHSDESDEL), Peptide2, OPN-ASARM^5S-A^ (RKRDDAHQADEAHHADEADEL), Peptide3, OPN-ASARM^3S-A^ (RKRDDSHQADESHHADEADEL), and Peptide4, OPN-ASARM^2S-A^ (RKRDDAHQSDEAHHSDESDEL).

FAM20C kinase assays with peptide substrates were performed in 10 µl reactions, with 500 µM ATP, including 3 µCi (0.05 µM) of [gamma-^32^P]ATP (6000 Ci/mmol, PerkinElmer), 50 mM Tris (pH 7.0), 10 mM MnCl2, 0.1% BSA, affinity purified FAM20C:V5 and peptides. Casein Kinase I (500 units, NEB) was used as a control kinase. Mutant versions (5 ng, 0.066 pmol), and peptides (2.5 µg, 1 nmol as a standard condition, 250 ng, 100 pmol in FAM20C mutants). Reactions were stopped by adding 1.5 µl 0.1N-HCl, and one half of the reaction volume was pipetted on to P81 phospho-cellulose filter paper [45]. The filter papers were washed with 0.5% phosphoric acid and subjected to liquid scintillation counting.

### Immunostaining

HEK293T cells were plated on a tissue culture slide (8 chamber, Thermo Fisher Scientific), and pcDNA-FAM20C:V5 or its mutant forms were transfected. For S2 cells, UASattB-CG31145:V5 or UASattB-CG3631:V5 and pWAGal4 were co-transfected in the tissue culture slide. 48 hours after transfection, the cells were fixed with 4% paraformaldehyde in PBS, and immuno-stained. Mouse anti-V5 (1∶500, Invitrogen), Rabbit anti-V5 (1∶500, Bethyl Laboratories), Rabbit anti-Giantin (1∶500, Abcam), Mouse anti-KDEL (1∶250, Stressgen), mouse anti-Drosophila Golgi (7H6D7C2, 1∶500, EMD Biosciences), and rabbit anti-Drosophila GM130 (1∶200, Abcam) were used as primary antibodies. Alexa Fluor 488-conjugated goat anti-mouse or anti-rabbit IgGs (1∶100, Invitrogen), and Cy3-conjugated anti-mouse or anti-rabbit IgGs (1∶100, Jackson Immuno Research Laboratories) were used as secondary antibodies. Images were obtained on a confocal microscope (FV-1000D, Olympus).

### Molecular Biology

Mouse FAM20C, from 98 bp upstream of the ATG to the last codon, was amplified from cDNA clone BC025826 (Open Biosystems) by PCR, using as primers CAAAGCTTGGACCTTGACCCGCGGGTCGTTG and AGCCGCGGCCGCCCCTCTCCGTGGAGGCTCTG, and cloned into HindIII and NotI cut pcDNA3.1/V5-HisB (Invitrogen) to create pcDNA-FAM20C:V5. Mouse FAM20A, from 91 bp upstream of the ATG to the last codon, was amplified from cDNA clone BC029169 (Open Biosystems) by PCR, using as primers GGAATTCTAATCCCCTGTGTGAGCATT and TTTGGCGGCCGCCGCTCGTCAGATTAGCCTGGC, and cloned into EcoRI and NotI cut pcDNA3.1/V5-HisB to create pcDNA-FAM20A:V5. Mouse FAM20B, from 57 bp upstream of the ATG to the last codon, was amplified from cDNA clone BC019381 (Open Biosystems) by PCR, using as primers CGAATTCTGTTCCCTGTGATAAGCCAG and GCATGCGGCCGCCCAAGTGGGAGAGTGGCATC, and the resulting fragments was cloned into EcoRI and NotI cut pcDNA3.1/V5-HisB to create pcDNA-FAM20B:V5.

Mouse OPN, from 73 bp upstream of the ATG to the last codon, was amplified from cDNA clone BC057858 (Open Biosystems) by PCR, using as primers AAGGTACCCATCCTTGCTTGGGTTTGCAG and GTTTTTCCGCGGCCGCCCGTTGACCTCAGAAGATGAAC, and cloned into KpnI and SacII cut pMT(WB)-Ds1–10:FLAG [22] to create pMT(WB)-OPN:FLAG. pMT(WB)-OPN:FLAG was digested with KpnI and AgeI and cloned into KpnI and AgeI cut pcDNA3.1/V5-HisB to create pcDNA-OPN:FLAG. Mouse BSP from 66 bp upstream of the ATG to the last codon, was amplified from cDNA clone BC045143 (Open Biosystems) by PCR, using as primers AAGGTACCGAGAACAATCCGTGCCACTC and GGGAGCGGCCGCCCCTGATGGTAGTAATAATTCTG, and cloned into KpnI and NotI cut pcDNA-OPN:FLAG to create pcDNA-BSP:FLAG. Mouse DMP1, from 39 bp upstream of the ATG to the last codon, was amplified from cDNA clone BC113753 (Open Biosystems) by PCR, using as primers AAGGTACCCCTTGGGAGCCAGAGAGGGTAG and ACAAGCGGCCGCCCGTAGCCGTCCTGACAGTCATTG, and cloned into KpnI and NotI cut pcDNA-OPN:FLAG to create pcDNA-DMP1:FLAG. Mouse DSPP, from 76 bp upstream of the ATG and to the last codon, was amplified from cDNA clone BC129802 (Open Biosystems) by PCR, using as primers AAGGTACCCCTGGAAAGAGAGATAAGGAAATC and TTCTGCGGCCGCCCATCATCACTGGTTGAGTGGTTAC, and cloned into KpnI and NotI cut pcDNA-OPN:FLAG to create pcDNA-DSPP:FLAG. Mouse MEPE, from 36 bp upstream of the ATG and to the last codon, was amplified from cDNA clone BC119162 (Open Biosystems) by PCR, using as primers AAGGTACCTCCTGAAGGTGAATGACGCCAG and AATCGCGGCCGCCCGTCACCATGACTCTCACTAG, and cloned into KpnI and NotI cut pcDNA-OPN:FLAG to create pcDNA-MEPE:FLAG.

CG31145, from the ATG to the last codon, was amplified from cDNA clone RE73615 by PCR, using as primers AATGGTACCATGGCCGTCCTGCGTACTATG and TTATCTAGACGAGGAGACGTCCGTCTCGGATC, and cloned into KpnI and XbaI cut pUASTattB-yki:V5 [46] and 50 bp upstream of the ATG was added by PCR by using primers CCGCGGCTCGAGGGTACCAAAAAGCCATTTCTGCTGCAAGCAACAACAGTTGCAACACCAATCCCATCATGGCCGTCCTG and CAGGACGGCCATGATGGGATTGGTGTTGCAACTGTTGTTGCTTGCAGCAGAAATGGCTTTTTGGTACCCTCGAGCCGCGG to create UASattB-CG31145:V5. CG3631, from the ATG to the last codon, was amplified from cDNA isolated from S2 cells by PCR, using as primers GGCGGTACCATGAACAAGCGCAGCGTCATCATC and CTGTCTAGACAGAGTTTTGAACATTTTGTC, and cloned into KpnI and XbaI cut pUASTattB-yki:V5 and 50 bp upstream of the ATG was added by PCR by using primers GGCTCGAGGGTACCCTGCGTATACGTAATATTAAAAATAGGCTAACGCCCGCCCAGGCTGCAGGATGAACAAGCGCAG and CTGCGCTTGTTCATCCTGCAGCCTGGGCGGGCGTTAGCCTATTTTTAATATTACGTATACGCAGGGTACCCTCGAGCC to create UASattB-CG3631:V5.

Site-specific mutagenesis was performed by PCR essentially as described [47], by using primers CATCCACTTGGGCGGCGGGCGCGGG and CCCGCGCCCGCCGCCCAAGTGGATG (FAM20C^DN473–474GG^), TGGGGAACATGGGTCGGCATCACTAC and GTAGTGATGCCGACCCATGTTCCCCA (FAM20C^D453G^), GCATGCCCTGTGTAGGAGGCCCGAC and GTCGGGCCTCCTACACAGGGCATGC (FAM20C^G374R^), CATGCCCTGTGTGAGAGGCCCGACCA and TGGTCGGGCCTCTCACACAGGGCATG (FAM20C^G374E^), ATCGAAGGATCCCGGGCGGCCTTCC and GGAAGGCCGCCCGGGATCCTTCGAT (FAM20C^L383R^), GCCCTGGACCGGTGGTTGCGCATAG and CTATGCGCAACCACCGGTCCAGGGC (FAM20C^R544W^), CACAACCCAGCCAACGATGCCTTACTG and CAGTAAGGCATCGTTGGCTGGGTTGTG (FAM20C^I241N^), CCATGAAGTCAAGGGGCACGCAGCTG and CAGCTGCGTGCCCCTTGACTTCATGG (FAM20C^G261R^), GATATGACCGTCTTTAATTTCCTCATGG and CCATGAGGAAATTAAAGACGGTCATATC (FAM20C^D446N^). All mutations (numbering above corresponds to mouse amino acid sequence) were confirmed by DNA sequencing.

## Supporting Information

Figure S1 **Sequence similarity among Golgi kinases.** Alignment of the kinase domains of Fj and FAM20 proteins from diverse species. Amino acid similarities are highlighted by coloring, conservation of key catalytic motifs common among all protein kinases, named after their consensus sequences in typical protein kinases: VAIK, E, HxDxxxxN and DFG, is indicated by bars and lettering above the alignments. Species abbreviations in names are as follows: Hsap: Homo sapiens; Xtro: Xenopus tropicalis; Drer: Danio rerio; Cint: Ciona intestinalis; Skow: Saccoglossus kowlakovski; Bflo: Branchiostoma floridae; Dmel: Drosophila melanogaster; Phum: Pediculus humanus; Isca: Ixodes scapularis; Spur: Strongylocentrotus purpuratus; Nvec: Nematostella vectensis; Cint: Ciona intestinalis; Amel: Apis mellifera; Cele: Caenorhabditis elegans.
(PDF)

Figure S2 **Localization of FAM20 proteins.** A–f Localization of the indicated V5-tagged FAM20 proteins (green) transfected into HEK293T cells (a–d) or S2 cells (e–f) as compared to Golgi markers (Giantin, a,c, or p120 Golgi, e,g, magenta) or an ER marker (anti-KDEL, magenta). g) Localization of FAM20C (green) in MC3T3 cells is clearly distinct from an ER marker (KDEL). Panels marked by prime symbols show single channels of the merged image to the left. h) Western blot showing that FAM20C:V5 expressed in HEK293T cells is detected in both lysate (L) and medium (M), FAM20A and FAM20B are only detected in cell lysate.
(TIF)

Figure S3 **Additional characterization of Kinase activity of FAM20C.** All panels show the results of kinase assays using dephosphorylated alpha casein as a substrate, and affinity purified FAM20C:V5 as the enzyme. a) Dependency of kinase activity on pH. b) Dependency of kinase activity on calcium concentration.
(TIF)

Figure S4 **Sequence of FAM20C and mutant isoforms.** a) Amino acid sequence of human FAM20C (NP_064608.2). Amino acids altered in human patients or by our site-specific mutagenesis are identified by colors (red, from patients, blue, in conserved motifs) and numbers above. b) Tabulation of the location of mutated amino acids, to account for differences in annotation due to different sequence entries of human FAM20C, and differences in size of human versus mouse FAM20C.
(TIF)

Figure S5 **Localization of FAM20C mutant isoforms.** Examples of localization of the indicated V5-tagged FAM20C mutant proteins (green) transfected into 293 cells, as compared to a Golgi markers (Giantin, a–h, red) or an ER marker (anti-KDEL, i–p, red).
(TIF)
