# Supplementary figures and images for: The Raine Syndrome Protein FAM20C Is a Golgi Kinase That Phosphorylates Bio-Mineralization Proteins
Source: PLoS One. 2012 Aug 10;7(8):e42988. doi: 10.1371/journal.pone.0042988 (PMC3416761; doi:10.1371/journal.pone.0042988)

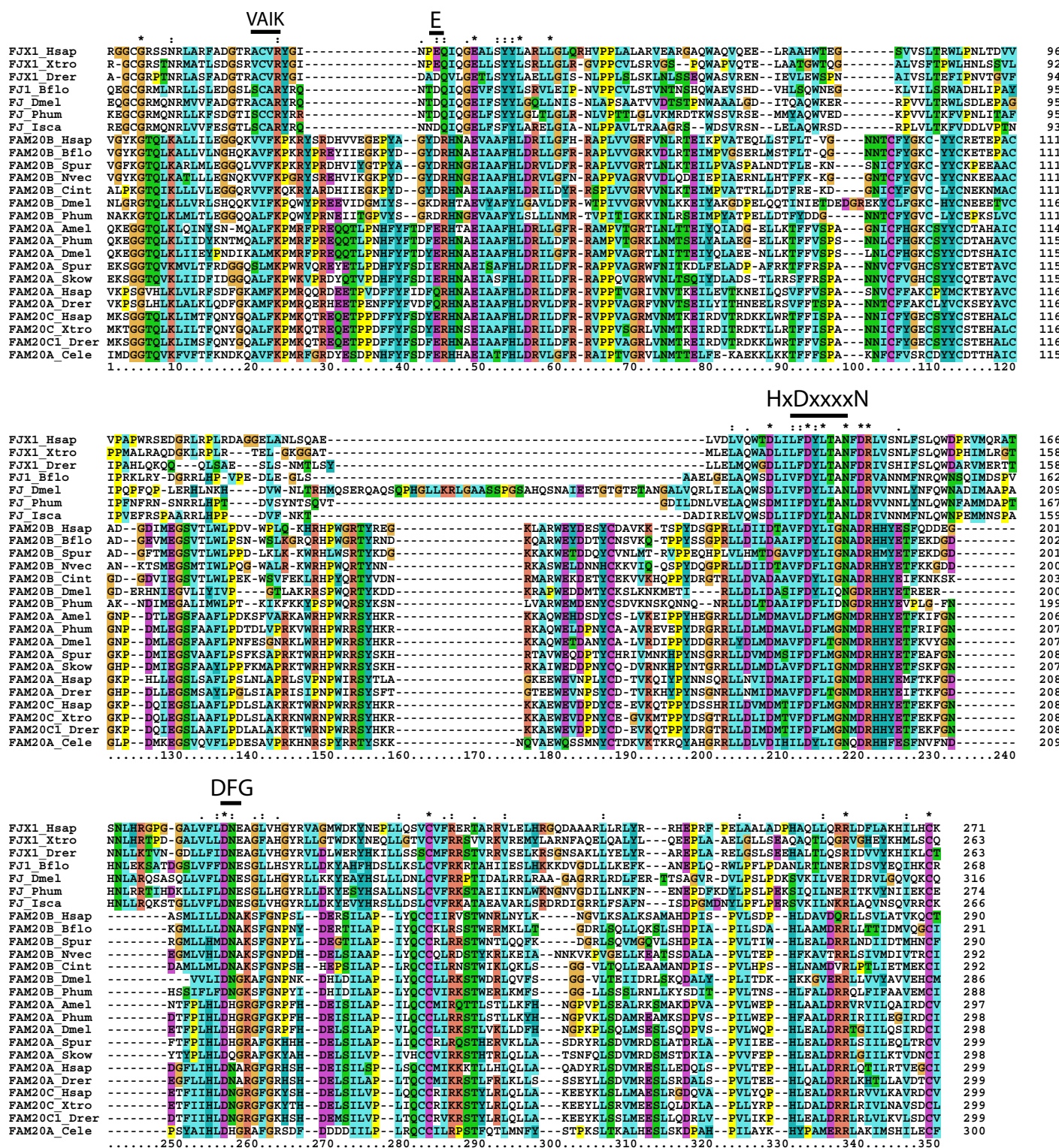

Supplement: Figure S1 — Sequence similarity among Golgi kinases. Alignment of the kinase domains of Fj and FAM20 proteins from diverse species. Amino acid similarities are highlighted by coloring, conservation of key catalytic motifs common among all protein kinases, named after their consensus sequences in typical protein kinases: VAIK, E, HxDxxxxN and DFG, is indicated by bars and lettering above the alignments. Species abbreviations in names are as follows: Hsap: Homo sapiens; Xtro: Xenopus tropicalis; Drer: Danio rerio; Cint: Ciona intestinalis; Skow: Saccoglossus kowlakovski; Bflo: Branchiostoma floridae; Dmel: Drosophila melanogaster; Phum: Pediculus humanus; Isca: Ixodes scapularis; Spur: Strongylocentrotus purpuratus; Nvec: Nematostella vectensis; Cint: Ciona intestinalis; Amel: Apis mellifera; Cele: Caenorhabditis elegans. (PDF) [file pone.0042988.s001.pdf]

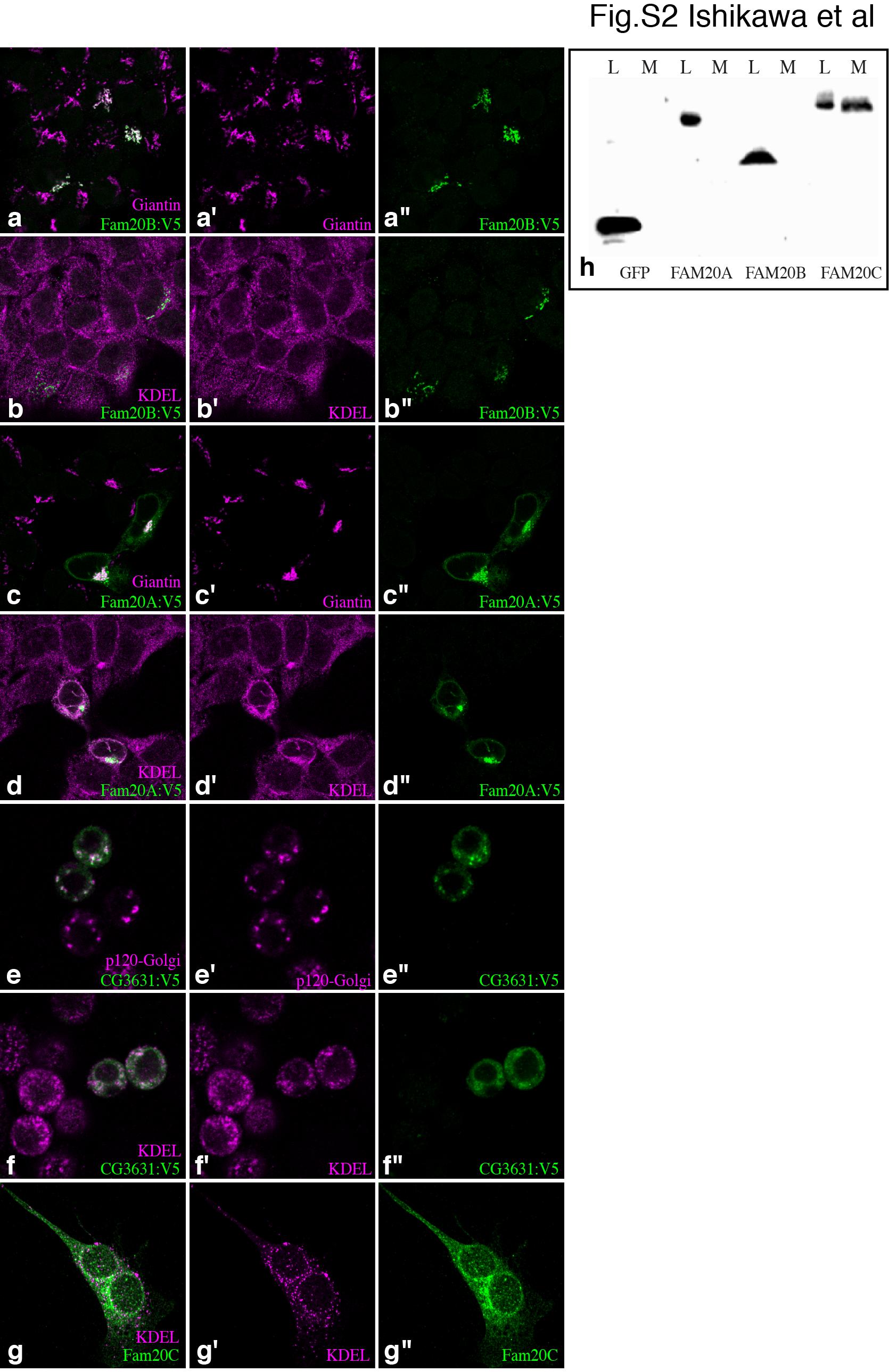

Supplement: Figure S2 — Localization of FAM20 proteins. A–f Localization of the indicated V5-tagged FAM20 proteins (green) transfected into HEK293T cells (a–d) or S2 cells (e–f) as compared to Golgi markers (Giantin, a,c, or p120 Golgi, e,g, magenta) or an ER marker (anti-KDEL, magenta). g) Localization of FAM20C (green) in MC3T3 cells is clearly distinct from an ER marker (KDEL). Panels marked by prime symbols show single channels of the merged image to the left. h) Western blot showing that FAM20C:V5 expressed in HEK293T cells is detected in both lysate (L) and medium (M), FAM20A and FAM20B are only detected in cell lysate. (TIF) [file pone.0042988.s002.tif]

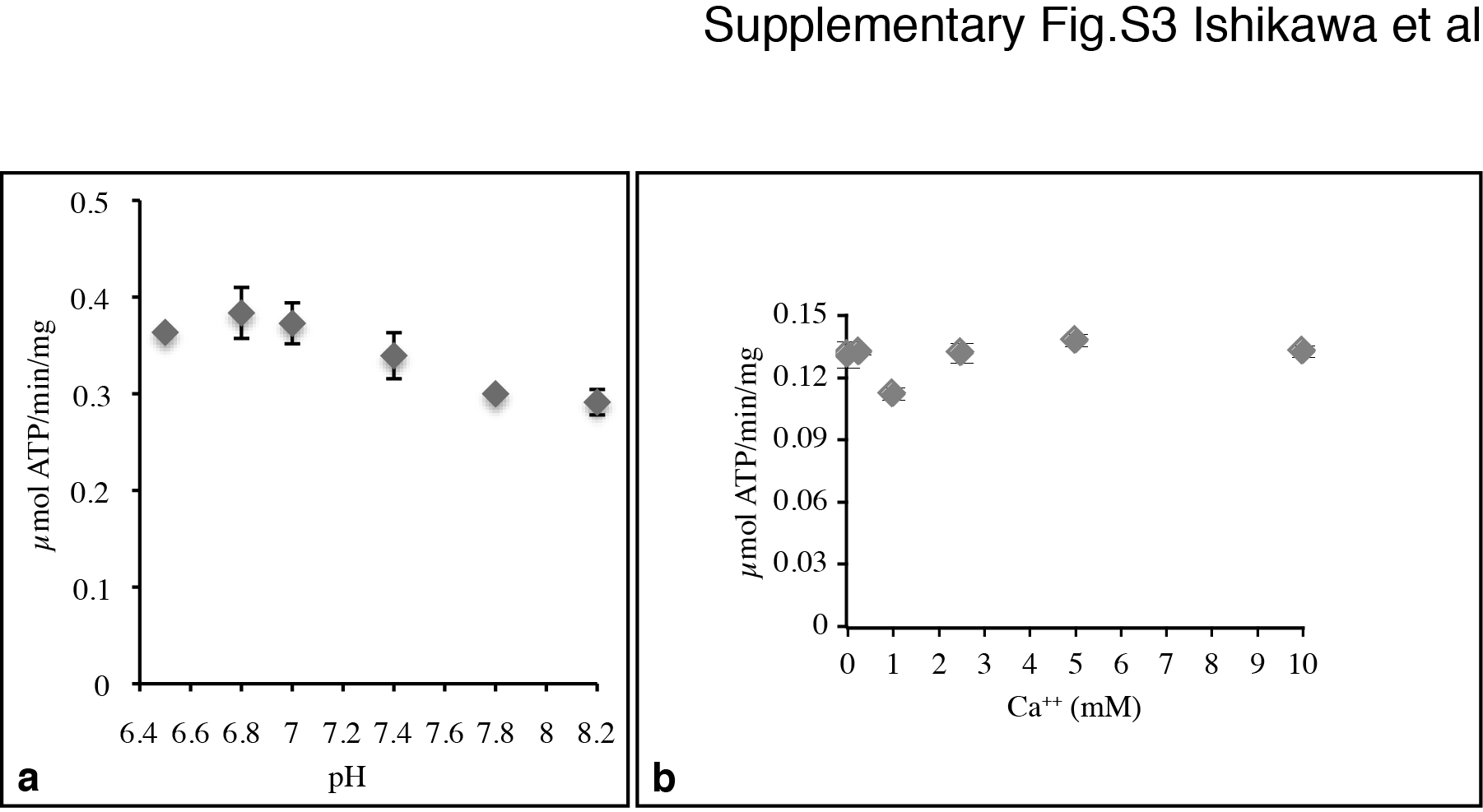

Supplement: Figure S3 — Additional characterization of Kinase activity of FAM20C. All panels show the results of kinase assays using dephosphorylated alpha casein as a substrate, and affinity purified FAM20C:V5 as the enzyme. a) Dependency of kinase activity on pH. b) Dependency of kinase activity on calcium concentration. (TIF) [file pone.0042988.s003.tif]

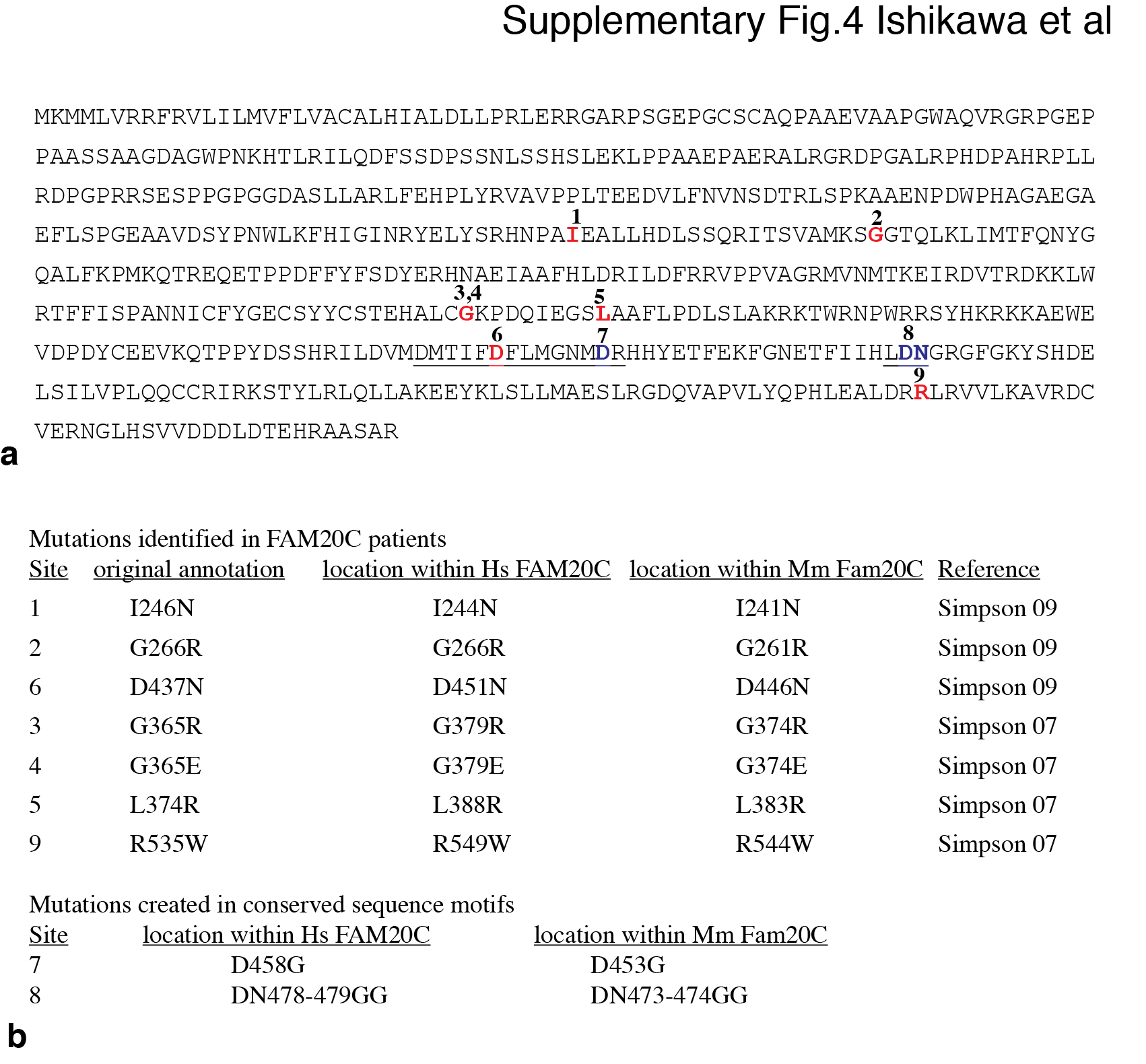

Supplement: Figure S4 — Sequence of FAM20C and mutant isoforms. a) Amino acid sequence of human FAM20C (NP_064608.2). Amino acids altered in human patients or by our site-specific mutagenesis are identified by colors (red, from patients, blue, in conserved motifs) and numbers above. b) Tabulation of the location of mutated amino acids, to account for differences in annotation due to different sequence entries of human FAM20C, and differences in size of human versus mouse FAM20C. (TIF) [file pone.0042988.s004.tif]

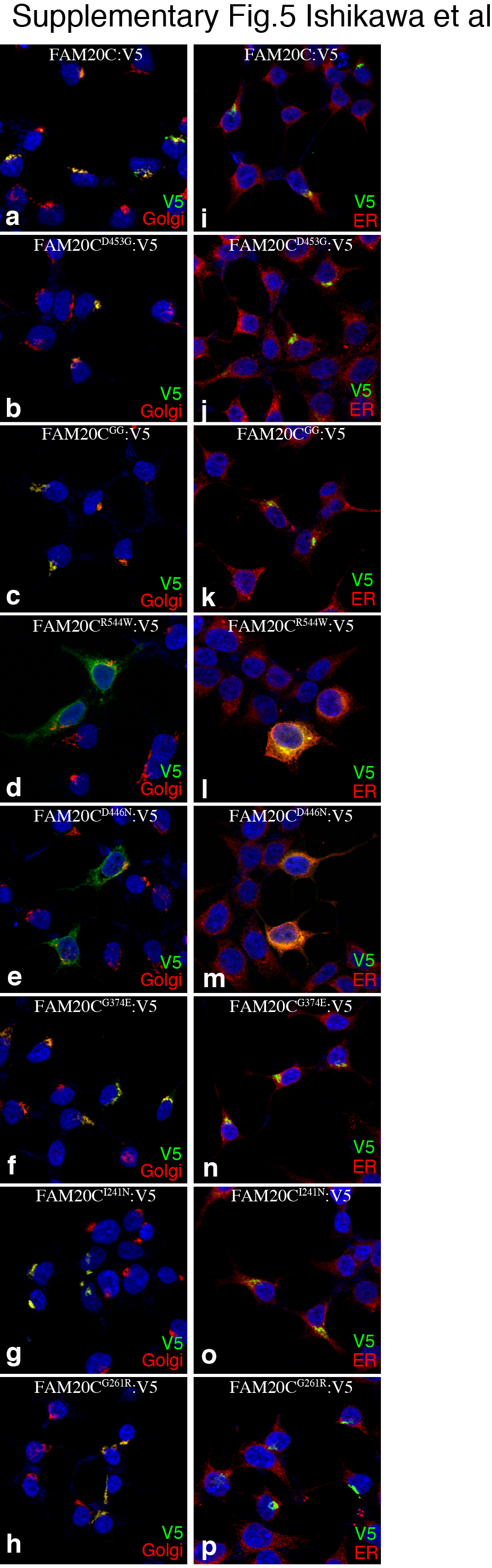

Supplement: Figure S5 — Localization of FAM20C mutant isoforms. Examples of localization of the indicated V5-tagged FAM20C mutant proteins (green) transfected into 293 cells, as compared to a Golgi markers (Giantin, a–h, red) or an ER marker (anti-KDEL, i–p, red). (TIF) [file pone.0042988.s005.tif]
